# Genome-Wide Association Study and Identification of Candidate Genes for Intramuscular Fat Fatty Acid Composition in Ningxiang Pigs

**DOI:** 10.3390/ani13203192

**Published:** 2023-10-13

**Authors:** Qinghua Zeng, Hu Gao, Shishu Yin, Yinglin Peng, Fang Yang, Yawei Fu, Xiaoxiao Deng, Yue Chen, Xiaohong Hou, Qian Wang, Zhao Jin, Gang Song, Jun He, Yulong Yin, Kang Xu

**Affiliations:** 1 Animal Nutrition Genome and Germplasm Innovation Research Center, College of Animal Science and Technology, Hunan Agricultural University, Changsha 410128, China; chuweixiang168168@163.com (Q.Z.); gaohu_20190008@163.com (H.G.); yinshishu2019@126.com (S.Y.); y621829@163.com (F.Y.); fuyw2020@163.com (Y.F.); wangq0130@163.com (Q.W.); jz2725552543@163.com (Z.J.); sg19971109@163.com (G.S.); hejun@hunau.edu.cn (J.H.); 2 Laboratory of Animal Nutrition Physiology and Metabolism, The Institute of Subtropical Agriculture, Chinese Academy of Sciences, Changsha 410125, China; 2017102040003@whu.edu.cn (X.D.); 18837025618@126.com (Y.C.); 17865669807@163.com (X.H.); 3 Hunan Institute of Animal & Veterinary Science, Changsha 410131, China; 13907487646@126.com; 4 Guangdong Laboratory for Lingnan Modern Agriculture, Guangzhou 510642, China

**Keywords:** *longissimus dorsi*, saturated fatty acids, monounsaturated fatty acids, polyunsaturated fatty acids, GWAS

## Abstract

**Simple Summary:**

In this study, we conducted a comprehensive investigation of the fatty acid composition in Ningxiang pigs using a genome-wide association study. Our findings revealed a combination of previously reported and novel candidate genes associated with saturated fatty acids (SFAs), monounsaturated fatty acids (MUFAs), and polyunsaturated fatty acids (PUFAs). Notably, we identified significant single-nucleotide polymorphisms (SNPs) that are closely linked to specific fatty acids, and some of these genes explained substantial phenotypic variance. These noteworthy discoveries have the potential to significantly improve meat quality and fat deposition in Ningxiang pigs through targeted breeding approaches. Our research provides valuable insights into the intricate composition of fatty acids, thus offering practical implications for elevating meat quality and ultimately benefiting both the pig industry and consumers. The significance of this study is underscored by its potential to drive positive changes in society by promoting healthier and superior-quality pork products.

**Abstract:**

Ningxiang pigs exhibit a diverse array of fatty acids, making them an intriguing model for exploring the genetic underpinnings of fatty acid metabolism. We conducted a genome-wide association study using a dataset comprising 50,697 single-nucleotide polymorphisms (SNPs) and samples from over 600 Ningxiang pigs. Our investigation yielded novel candidate genes linked to five saturated fatty acids (SFAs), four monounsaturated fatty acids (MUFAs), and five polyunsaturated fatty acids (PUFAs). Significant associations with SFAs, MUFAs, and PUFAs were found for 37, 21, and 16 SNPs, respectively. Notably, some SNPs have significant PVE, such as ALGA0047587, which can explain 89.85% variation in Arachidic acid (C20:0); H3GA0046208 and DRGA0016063 can explain a total of 76.76% variation in Elaidic Acid (C18:1n-9(t)), and the significant SNP ALGA0031262 of Arachidonic acid (C20:4n-6) can explain 31.76% of the variation. Several significant SNPs were positioned proximally to previously reported genes. In total, we identified 11 candidate genes (*hnRNPU*, *CEPT1*, *ATP1B1*, *DPT*, *DKK1*, *PRKG1*, *EXT2*, *MEF2C*, *IL17RA*, *ITGA1* and *ALOX5*), six candidate genes (*ALOX5AP*, *MEDAG*, *ISL1*, *RXRB*, *CRY1*, and *CDKAL1*), and five candidate genes (*NDUFA4L2*, *SLC16A7*, *OTUB1*, *EIF4E* and *ROBO2*) associated with SFAs, MUFAs, and PUFAs, respectively. These findings hold great promise for advancing breeding strategies aimed at optimizing meat quality and enhancing lipid metabolism within the intramuscular fat (IMF) of Ningxiang pigs.

## 1. Introduction

Ningxiang is a famous breed of pig in China, with a history spanning more than 1000 years. As an obese breed, Ningxiang is superior to lean meat breeds in terms of intramuscular fat (IMF). Its market weight is approximately 74 kg, and the carcass slaughter rate is approximately 74 percent [1]. Ningxiang pork products are popular due to their unique flavor and nutritional value. Research has shown that the flavor and nutritional value of meat are closely related to the IMF content and fatty acid composition [2,3,4].

Fatty acids play a crucial role in the flavor of pork as fat-soluble flavor precursors [5,6]. Oleic acid (C18:1) is a major monounsaturated fatty acid (MUFA) in the fatty acid composition and is the most abundant, accounting for approximately 40% of the total fatty acid content. C18:1 in beef fat provides the meat with good tenderness, flavor, and antioxidant capacity [7,8]. Moreover, the type and content of fatty acids in meat diets can impact human health. For example, linoleic acid aids in slowing the progression of atherosclerosis [9]. Furthermore, the ratios of n-3, n-6, and other polyunsaturated fatty acids (PUFAs) are closely related to human diseases, such as cardiovascular disease and depression, as well as growth and development [10,11,12,13]. In summary, the fatty acid profile of pork is a crucial evaluation criterion for meat quality.

Genome-wide association studies (GWAS) can efficiently and accurately identify candidate genes related to target traits using single nucleotide polymorphisms (SNPs) as genetic molecular markers. In recent years, researchers have discovered numerous candidate genes associated with porcine IMF deposition and fatty acid composition through GWAS. A GWAS of the IMF in the Italian White breed by Davoli et al. revealed seven new SNPs, and three new candidate genes were annotated. These genes were not related to the IMF content [14]. Van et al. [15] used data from the Axiom pig 660K array to conduct a GWAS on the IMF of 454 Duroc and 659 Landrace boars and identified two quantitative trait locus (QTL) regions for newly synthesized fatty acid traits on SSC4 and SSC14 in Duroc pigs. Viterbo et al. [16] performed a GWAS on the fatty acid composition of 480 purebred Duroc pigs, identifying 25, 29, and 16 SNP loci significantly associated with stearic acid, oleic acid, and saturated fatty acids (SFAs), respectively. The genetic factors contributing to the fatty acid composition of Ningxiang pigs remain unknown. This study aimed to examine significant candidate genes for specific fatty acid compositions and explore potential biological pathways by conducting a GWAS on the fatty acid composition of Ningxiang pigs.

## 2. Materials and Methods

### 2.1. Animal Harvest and Sample Collection

The feeding and dietary conditions of all Ningxiang pigs were the same, and samples were collected in two batches (July 2019 and August 2020). *Longissimus dorsi* samples (taken from the 12th ribs) were collected from 691 Ningxiang pigs that were slaughtered at a predetermined age (180 ± 5 days age) at the Ningxiang Chu Weixiang Slaughterhouse and Meat Processing, LLC (Ningxiang City, Hunan Province, China). A sample of approximately 2 × 1 × 1 cubic centimeters was quickly taken and stored in a self-sealing bag with dry ice for IMF measurement. Additionally, the samples used for DNA extraction were simultaneously stored in a liquid nitrogen tank.

### 2.2. Determining Intramuscular Fat and Fatty Acids

In this study, we used Soxhlet extraction to measure the IMF contents of the 691 *longissimus dorsi* samples according to the standard “Meat and Meat Products-Determination of Free Fat Content” (GB/T 9695.1-2008) [17]. The composition of fatty acids was determined via gas chromatography (GC), with the specific procedure detailed below. First, 0.5 g of sample powder was accurately weighed, dried to a constant weight and placed in a 10 mL centrifuge tube. Next, 2 mL of a benzene and petroleum ether mixture (1:1 by volume) was added, and the tube was wrapped in tin foil and left in a dark place for 24 h. Then, 2 mL of a 0.4 mol/L KOH methanol solution was added for methylation. The sample was shaken well and left to stand for 15 min. Double-distilled water was added to a volume of 10 mL, and the sample was centrifuged at 10,000 rpm for 10 min to obtain 100 µL of supernatant. The supernatant was finally diluted with hexane. A gas chromatograph (Agilent 7890A, Santa Clara, CA, USA) was used to determine the content of medium- to long-chain fatty acids. The GC analysis conditions were as follows: the chromatographic column was an SP-2560 (100 m × 0.25 mm, 0.20 µm) capillary column, and high-purity nitrogen was used as the carrier gas. The heating program was as follows: initial temperature of 140 °C maintained for 5 min, then increased to 240 °C at 4 °C/min; sample inlet temperature of 260 °C; flame ionization detector (FID) temperature of 260 °C; split ratio of 100:1; and injection volume of 1 µL. A total of 25 fatty acids were detected (Table S1), and only those present in more than 80% of individuals (N ≥ 533) were retained (Table 1).

### 2.3. DNA Extraction, Genotyping and Quality Control

#### 2.3.1. DNA Extraction and Genotyping

Genomic DNA was extracted from muscle tissue using the standard phenol-chloroform method, and the DNA was dissolved in TE buffer. The concentration and purity of the DNA samples were measured using a Nanodrop One spectrophotometer (Thermo Fisher Scientific, Waltham, MA, USA). Samples with an A_260/280_ ratio of 1.7~2.0 were genotyped using the GeneSeek Genomic Profiling (GGP) version 2 Porcine 50K SNP chip (Neogen Corporation, Lincoln, NE, USA), which comprises 50,697 SNP loci.

#### 2.3.2. Quality Control and Genotype Imputation

**Figure 1:**
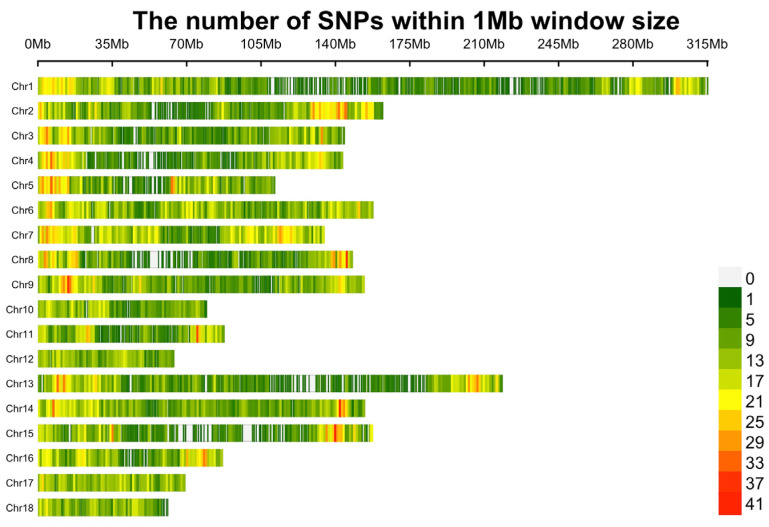
Distribution plot of SNPs after quality control.

First, SNPs located on the X and Y chromosomes and unknown or duplicate locations were removed. To decrease the missing genotype rate, Beagle 5.1 software [18] was employed to impute the remaining 38,817 SNPs. Then, quality control was conducted using PLINK v1.9 [19] with the following criteria: (1) SNP call rate ≥ 90%; (2) minor allele frequency (MAF) ≥ 1%; and (3) Hardy–Weinberg equilibrium (HWE) testing *p* value ≥ 10^−6^. After quality control, 12,201 SNPs were removed due to missing genotype rates, MAFs, and HWE. Finally, 691 individuals and 25,809 SNPs remained for subsequent analysis (Figure 1).

### 2.4. Population Stratification

**Figure 2:**
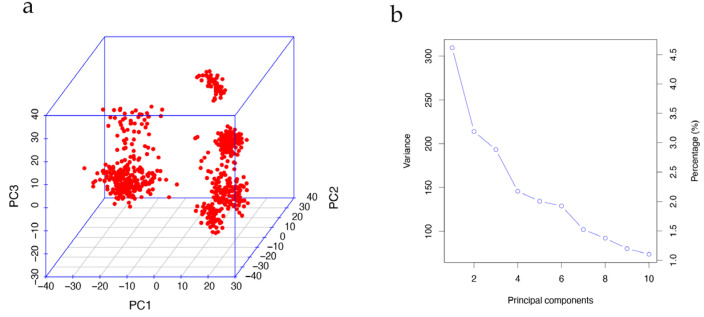
Principal component analysis. (**a**) Visualization of the first three PC values showing the existence of population stratification. (**b**) Screen plot of the first 10 PC values. Decreasing trends indicate that the first five PCs can be appropriately used to correct the population stratification.

To mitigate the risk of concealed population stratification leading to spurious results in the GWAS, we conducted principal component analysis (PCA) using imputed genotypes (25,809 SNPs) with PLINK v1.9 (command: --pca). As depicted in Figure 2a, the population exhibited significant population stratification, necessitating the incorporation of the first principal components (PCs) for correction.

### 2.5. Genome-Wide Association Study

The association between SNPs and fatty acids was examined using the BLINK model (Bayesian-information and Linkage-disequilibrium Iteratively Nested Keyway) using the GAPIT 3.0 package [20]. BLINK, an enhanced version of FarmCPU, enhances statistical power by relaxing the assumption of even distribution of trait-related genes across the genome and incorporates the Bayesian information criterion (BIC) in fixed effects models to improve computational efficiency [21]. The model integrates Equations (1)–(3) according to the BIC strategy, iteratively calculating and excluding all pseudo-quantitative trait nucleotides (QTNs) to identify significant loci.

(1) y=Xa+Zb+e

(2) y=Xa+Zb+Qp+e

(3) y=Xa+Qp+e

where *y* is a vector of phenotypic data; *a* is the vector of fixed effects or covariates, including IMF content and the first five PCs; *b* is a vector of marker effects; *p* is the effect of pseudo-QTNs; *X*, *Z*, and *Q* are the incidence matrices corresponding to *a*, *b*, and *p*, respectively; and *e* is the vector of residual errors. The BLINK model uses Equation (1) to define pseudo-QTNs as a covariate for Equation (2). The SNP obtained from Equation (2) determines the information of QTNs according to linkage disequilibrium (LD) and then employs Equation (3) to perform accuracy detection of QTNs using the BIC strategy. The false discovery rate (FDR) method of multiple testing, as described by Benjamini-Hochberg, was utilized to measure the statistical significance of association studies at a genome-wide level. The cut-off for considering SNPs as significant was set at FDR ≤ 0.1. The phenotypic variance explained (*PVE*) by genetic effects was calculated as follows [22]:

(4) $$PVE=\frac{2\alpha^{2}MAF(1-MAF)}{2\alpha^{2}MAF(1-MAF)+(se(\alpha^{2})2N\times MAF(1-MAF)}$$

where *MAF* is the minor allele frequency for the SNP, $\alpha^{2}$ is the effect of the SNP marker, *N* is the sample size, and $se(\alpha^{2})$ is the standard error of $\alpha^{2}$.

### 2.6. Estimation of Heritability and Genetic Correlation

Heritability (*h*^2^) was estimated using the following Formula (5) in HIBLUP [23]:

(5) $$h^{2}=\frac{\sigma_{a}^{2}}{\sigma_{a}^{2}+\sigma_{e}^{2}}$$

where $\sigma_{a}^{2}$ and $\sigma_{e}^{2}$ are the additive genetic variance and the residual variance, respectively. The phenotypic and genetic correlation (*r_p_* and *r_g_*) between IMF and FAs were estimated using HIBLUP software [23].

(6) $$r_{P}=\frac{{COV}_{P_{xy}}}{\sqrt{\sigma_{P_{x}}^{2}\times \sigma_{P_{y}}^{2}}};\;r_{G}=\frac{{COV}_{G_{xy}}}{\sqrt{\sigma_{G_{x}}^{2}\times \sigma_{G_{y}}^{2}}}$$

where ${COV}_{P_{xy}}$ and ${COV}_{G_{xy}}$ represent the phenotypic and genetic covariance, respectively. $\sigma_{P}^{2}$ and $\sigma_{G}^{2}$ are the phenotypic and genetic variance, respectively.

### 2.7. Identification of Candidate Genes

Candidate genes were identified based on their physical positions and functions according to the Sus scrofa 10.2 reference genome assembly. The SNP-containing or nearest annotated genes for each potential SNP were obtained from the Sus scrofa (10.2) gtf file (http://ftp.ensembl.org/pub/release-80/gtf/sus_scrofa/Sus_scrofa.Sscrofa10.2.80.gtf.gz accessed on 1 May 2015) and taken as candidate genes.

### 2.8. Functional Enrichment Analysis

The g:Profiler website [24] was used for Gene Ontology (GO) and Kyoto Encyclopedia of Genes and Genomes (KEGG) enrichment analysis. A tailor-made algorithm was chosen for multiple testing correction (adjusted *p* value < 0.05).

## 3. Results

### 3.1. Descriptive Statistics for IMF and Fatty Acid Composition

The statistical analysis results of IMF and 25 fatty acids are presented in Table S1. The IMF content in the *longissimus dorsi* of Ningxiang pigs was determined to be 3.65%. In the overall fatty acid distribution, MUFAs had the highest proportion at 41.88%, followed by SFAs at 39.35%, and PUFAs at 12.78%. Eleven fatty acids, including C22:6n-3, C17:1, and C15:0, were removed due to a sample size below 553 individuals (<80%). The remaining 14 fatty acids and IMF were used for subsequent analysis (Table 1). Among these fatty acids, oleic acid (C18:1n-9(c)) exhibited the highest content (40.28%), while elaidic acid (C18:1n-9(t)) had the lowest content (0.11%).

### 3.2. Estimation of Genetic Parameters

**Figure 3:**
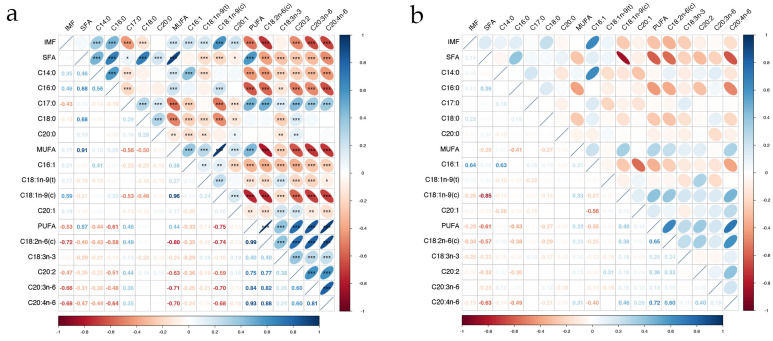
Correlations among IMF and 14 fatty acids in the *longissimus dorsi* of Ningxiang pigs. “***” represents *p* value < 0.001, “**” represents *p* value < 0.01, “*” represents *p* value < 0.05. (**a**) Phenotypic correlation; (**b**) genetic correlation.

The results of the correlation analysis between fatty acids and IMF are presented in Figure 3a,b. In the phenotypic correlation analysis, the majority of fatty acids, except for SFA, C20:0, and C18:3n-3, exhibited a significant correlation with IMF (*p* < 0.001). SFA demonstrated significant positive phenotypic correlations with MUFAs and PUFAs (*p* < 0.001) but showed negative genetic correlations with four MUFAs and five PUFAs (*p* < 0.05). Furthermore, SFA, MUFA, and PUFA were significantly positively correlated with five SFAs, four MUFAs, and five PUFAs, respectively (*p* < 0.05). In the genetic correlation analysis, C16:1 had the highest correlation with IMF *(r_g_* = 0.64), and SFA was negatively correlated with most MUFAs and PUFAs, except C16:1 and C18:1n-9(t). All fatty acids had moderate to high heritabilities (0.37~0.89). Fatty acids C17:0 and C18:1n-9(t) had the lowest heritability estimates (0.45 and 0.37, respectively). Most fatty acids exhibited heritability estimates above 0.6. Notably, C18:0 exhibited the highest heritability estimate of 0.89 (Table 1).

### 3.3. Genome-Wide Association Study Results for Fatty Acids

After quality control, 25,809 SNPs for 691 Ningxiang pigs were retained for the GWAS. In total, 74 genome-wide level SNPs were identified for 14 FAs in this study.

#### 3.3.1. SFA

Thirty-seven genome-wide significant SNPs were identified for five SFAs (C14:0, C16:0, C17:0, C18:0, and C20:0). C20:0 had the most loci, which were located on SSC2, SSC3, SSC4, SSC5, SSC7, SSC8, SSC13, SSC14, and SSC16 (Figure S1). Moreover, some loci, such as ALGA0047587 (89.85%), ASGA0059505 (11.39%), and DRGA0011206 (6.85%) (Table S2), explained a large portion of the phenotypic variance.

#### 3.3.2. MUFA

The Manhattan plots showed that 21 genome-wide significant loci were identified on 12 chromosomes (SSC1, SSC2, SSC3, SSC4, SSC5, SSC7, SSC9, SSC11, SSC13, SSC14, SSC16, and SSC17) for four MUFAs (Figure S2). H3GA0046208 explained the largest portion of the phenotypic variance (45.24%) for C18:1n-9(t) (Table S2).

#### 3.3.3. PUFA

The Manhattan plots showed that 16 genome-wide significant loci on seven chromosomes (SSC1, SSC2, SSC5, SSC8, SSC9, SSC13, and SSC16) were identified for five PUFAs (Figure S3).

### 3.4. Identification of Candidate Genes

Four hundred and fifty-three genes were identified within a 500 kb region upstream and downstream of the significant SNPs (Table S2). For SFAs, 354 genes were found in a 1 Mb genomic region; 40 genes were close to 37 loci, of which 11 SNPs (WU_10.2_3_116903421, ASGA0074106, WU_10.2_4_119395133, M1GA0024654, WU_10.2_3_142168876, ALGA0010606, WU_10.2_11_3591593, ALGA0080940, H3GA0041501, MARC0054269, and WU_10.2_16_59778879) were located within 11 genes (*ALK*, *MFAP3*, *CEPT1*, *NPEPPS*, *ENSSSCG00000008655*, *SWI5*, *CDK8*, *AVPI1*, *ALOX5*, *ITGA1*, and *SLIT3*). For MUFAs, six SNPs (ALGA0015731, ASGA0064960, H3GA0025990, H3GA0046208, WU_10.2_5_13180559, and ASGA0031521) were intragenic variants. For PUFAs, 16 SNP loci were identified in 16 genes.

### 3.5. Functional Enrichment of Candidate Genes

**Figure 4:**
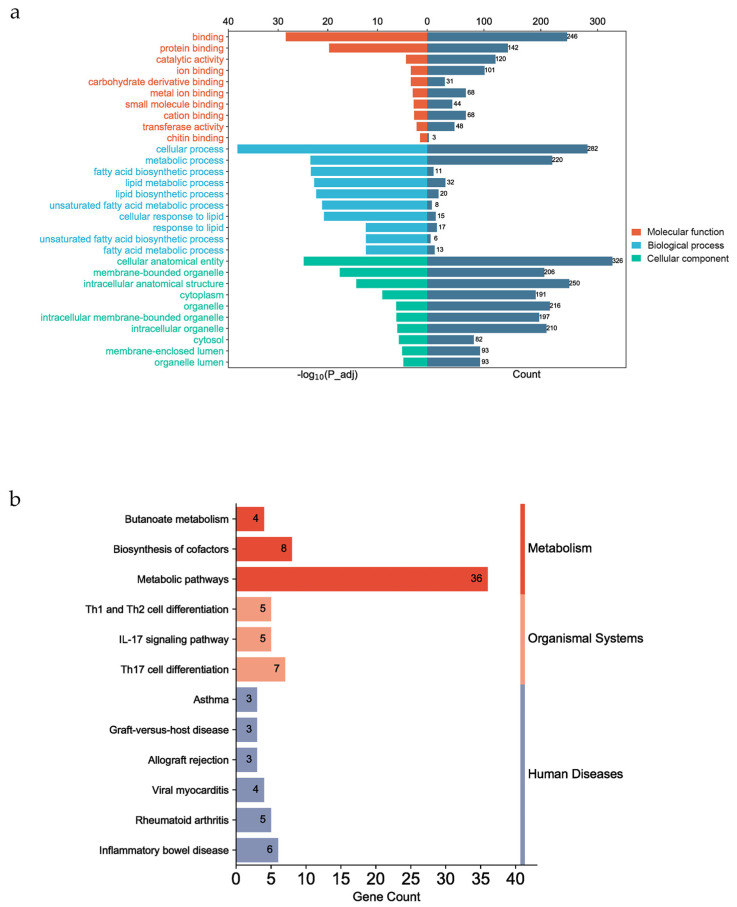
Gene functional enrichment analysis. (**a**) The top 10 enriched molecular functions and cellular components and 10 biological processes associated with fatty acid and lipid metabolism. (**b**) The 12 enriched KEGG pathways, which can be divided into three categories on the KEGG website (Metabolism, Organismal Systems, and Human Diseases).

The GO and KEGG enrichment analyses were performed using the g:Profiler website for all fatty acid traits. The functional genes were significantly enriched in 262 GO terms (p_adj < 0.05) (see File S1). The top 10 molecular functions were involved in binding. The top 10 cellular components were cellular anatomical entities (GO:0110165) and membrane-bound organelles (GO:0043227). There were some GO terms associated with lipid and fatty acid metabolism in the biological process category (Figure 4a), such as fatty acid biosynthetic process (GO:0006633), unsaturated fatty acid metabolic process (GO:0033559), lipid biosynthetic process (GO:0008610), and lipid metabolic process (GO:0006629). In this study, annotated genes were significantly enriched in 12 KEGG pathways, such as metabolic pathways (KEGG:01100), inflammatory bowel disease (KEGG:05321), and Th17 cell differentiation (KEGG:04659) (Figure 4b). These KEGG pathways can be categorized into three groups on the KEGG website: Metabolism, Organismal Systems, and Human Diseases (https://www.kegg.jp/kegg/pathway.html (accessed on 1 October 2023)).

## 4. Discussion

### 4.1. Phenotypic and Genetic Correlations

In this study, a total of 25 fatty acid species were detected in the *longissimus dorsi* of Ningxiang pigs, with 14 species commonly found in the population. SFA was the most abundant among them, while the MUFA relative content was the highest. According to research, the distribution pattern of fatty acids in different varieties of pork is similar [25]. However, there are significant differences in the composition of fatty acids in different tissues [26], which is also influenced by sex [27]. Interestingly, docosahexaenoic acid (DHA) has only been found in the tissues of Ningxiang pigs [26,28], but not in any other pig breeds or tissues [29,30]. DHA belongs to the omega-3 family alongside alpha-linolenic acid (ALA) and eicosapentaenoic acid (EPA), which are essential nutrients integral to human life. Both linoleic acid (*h*^2^ = 0.88) and eicosa-11,14-dienoic acid (*h*^2^ = 0.63) from the omega-3 family exhibit high heritability in the *longissimus dorsi* of Ningxiang pigs, which is an interesting phenomenon [31]. To ensure the safety and health of pork products, pig industry breeders and researchers have long sought to enhance the PUFA content and distribution in pork [32,33].

As an important indicator of pork quality, IMF has consistently garnered significant attention. Numerous studies have provided evidence for the impact of IMF on meat quality [34]. In recent years, the hypothesis that IMF deposition in muscle is impacted by fatty acid structure was verified [35]. By conducting a correlation analysis between IMF and various fatty acid components in the *longissimus dorsi* of Ningxiang pigs (Figure 3), it was observed that IMF exhibits a notably low correlation with SFAs. Furthermore, IMF exhibited a significant positive correlation with MUFAs and a significant negative correlation with PUFAs. Realini et al. [36] investigated the relationship between IMF deposition and fatty acid composition in New Zealand sheep and found results consistent with those of our study, revealing a negative correlation between MUFAs and IMF deposition, a positive correlation between PUFAs and IMF deposition, and a correlation coefficient of −0.72 between linoleic acid and IMF deposition. This phenomenon might be attributed to the endogenous synthesis rate and desaturation sequence of SFAs.

### 4.2. Candidate Genes for Fatty Acid Composition

Currently, the molecular mechanisms underlying fat deposition are a topic of interest. Fat deposition not only directly impacts the growth, development, and meat production traits of animals but also holds valuable implications for addressing human diseases. Ningxiang pigs are an excellent model for obesity research, and an increasing number of genes, regulatory factors, and metabolic pathways related to fat deposition in Ningxiang pigs have been identified [37]. In this study, we performed a comprehensive GWAS focusing on 14 fatty acids in the *longissimus dorsi* of Ningxiang pigs, and most of them exhibited significant correlations with IMF content. Seventy-four genome-wide significant SNPs were identified in this study, most of which were intronic and intergenic variations (Table S2). Only WU_10.2_2_9630034 is a missense mutation, and there is no research on the gene (*SDHAF2*) related to fatty acids.

A total of 40 genes were identified from 37 significant loci associated with SFAs which included *hnRNPU* [38], *CEPT1* [39], *ATP1B1*, *DPT*, *DKK1* [40], *PRKG1*, *EXT2* [41], *MEF2C*, *IL17RA* [42], *ITGA1* [43] and *ALOX5*. Among these, heteronuclear heterogeneous ribonucleoprotein particles (hnRNPs) represent a group of proteins with diverse functions, playing pivotal roles in RNA biogenesis, cellular localization, and transport [44]. Specifically, hnRNP U is involved in the *Blnc1/hnRNPU/EBF2* heterogeneous ribonucleoprotein particle complex, thus promoting the expression of brown adipocyte genes [38]. Additionally, Dickkopf WNT signaling pathway inhibitor 1 (*DKK1*) can regulate placental lipid metabolism through the WNT signaling pathway [40]. Surprisingly, muscle cell enhancer factor 2C (*MEF2C*) not only plays a crucial regulatory role in skeletal muscle cells but also exerts significant regulatory effects on adipose tissue deposition [45]. Elias et al. found that both *ALOX5* and *ALOX5AP* are involved in the browning of white adipose tissue through lipotoxin A4 [46]. In our study, arachidonic acid 5-nenenebb lipoxygenase (*ALOX5*) and its partner, arachidonic acid 5-lipoxygenase activator protein (*ALOX5AP*) were genes near the C20:0 and C16:1 significant SNP loci, respectively. In Ossabaw pig epicardial adipose tissue, *ALOX5* is positively correlated with n-3 PUFAs [47].

Twenty-five genes were annotated in the upstream and downstream regions of twenty-one loci related to MUFAs. Among them, genes such as *ALOX5AP* [46], *MEDAG* [48], *ISL1* [49], *RXRB* [50], *CRY1* [51], and *CDKAL1* [52] were found to be related to lipid synthesis and metabolism. Interestingly, *ALOX5AP* and *MEDAG* are two genes upstream and downstream of the MARC0099145 locus. Additionally, *MEDAG* is involved in fat metabolism, playing a crucial role in backfat deposition in most Western pig breeds [48]. Retinol-X receptor β (*RXRB*) is a member of the nuclear receptor superfamily of retinoic acid X receptors (RXRs) and is expressed in almost all tissues. In the liver, the activation of *RXRB* leads to increased expression of stearyl CoA desaturation (*SCD*) and *CD36* fatty acid transferase [50], *RXRB* is consistent with the results of this study and a study on the black Iberian pig [53]. Cryptochrome gene 1 (*CRY1*) is a member of the circadian clock gene family and plays a vital role in adipocyte biology. *CRY1* is regulated by the classic Wnt/β-catenin signaling pathway, which influences fat differentiation [54]. Moreover, *CRY1* contains two interaction regions that regulate its degradation to achieve diurnal blood glucose control [55], further affecting conditions such as obesity [51,56].

Nineteen genes were annotated in the upstream and downstream regions of 16 loci related to PUFAs. Genes such as *NDUFA4L2*, *SLC16A7*, *OTUB1*, *EIF4E* [57], and *ROBO2* [58] were found to be associated with lipid synthesis and metabolism. Mitochondrial dysfunction can cause an increase in *NDUFA4L2* expression, leading to lipid accumulation in renal cells [59]. In addition, *ssc-mir-708* is associated with fatty acids, but *miR-708* has only been reported in the regulation of cardiomyocyte proliferation to date [60]. Finally, eukaryotic translation initiation factor 4E (*EIF4E*) enhances the translation of various messenger RNAs involved in lipid metabolism processing and storage pathways, leading to weight gain after a high-fat diet [57]. These genes may influence the fatty acid composition. The majority of the fatty acids in Ningxiang pigs exhibit medium to high heritability, indicating that candidate genes are likely to impact the fatty acid composition of the *longissimus dorsi*. Therefore, the candidate genes related to fatty acids identified in this study can be considered for use in enhancing the fatty acid composition of imported or commercial pig meat, thereby improving the meat quality of commercial pigs.

## 5. Conclusions

This GWAS identified 74 genome-wide SNPs associated with 14 fatty acids in the *longissimus dorsi*. Some SNPs were located within or near reported genes, but some were novel for fatty acid composition. In total, twenty-two genes, such as *hnRNPU*, *ALOX5AP*, and *NDUFA4L2*, can be used as candidate genes for fatty acid composition in Ningxiang pigs. Our findings will be helpful for understanding the genetic basis of fatty acid composition and providing new targets for further breeding of pigs.

## Figures and Tables

**Table 1:** Detailed information on 14 fatty acids present in the IMF of the *longissimus dorsi* of Ningxiang pigs.

Fatty Acid	N	Relative Content (±SE) (%)	*h*^2^ (±SE)
IMF (Intramuscular fat)	691	3.65 (±0.04)	0.72 (±0.02)
SFA (Saturated fatty acid)	691	39.35 (±0.38)	0.61 (±0.04)
C14:0 (Myristic acid)	649	1.42 (±0.01)	0.80 (±0.03)
C16:0 (Palmitic acid)	651	26.38 (±0.06)	0.76 (±0.03)
C17:0 (Margaric acid)	645	0.15 (±0.001)	0.45 (±0.08)
C18:0 (Stearic acid)	651	13.42 (±0.06)	0.89 (±0.02)
C20:0 (Arachidic acid)	649	0.21 (±0.002)	0.87 (±0.04)
MUFA (Monounsaturated fatty acid)	691	41.88 (±0.42)	0.84 (±0.02)
C16:1 (Palmitoleic acid)	650	3.28 (±0.40)	0.87 (±0.03)
C18:1n-9(t) (Elaidic acid)	627	0.11 (±0.001)	0.37 (±0.09)
C18:1n-9(c) (Oleic acid)	651	40.28 (±0.12)	0.74 (±0.03)
C20:1 (Eicosenoic acid)	650	0.80 (±0.01)	0.78 (±0.03)
PUFA (Polyunsaturated fatty acid)	691	12.78 (±0.18)	0.60 (±0.03)
C18:2n-6(c) (Linoleic acid)	651	10.12 (±0.10)	0.61 (±0.03)
C18:3n-3 (α-Linolenic acid: ALA)	572	0.36 (±0.01)	0.63 (±0.08)
C20:2 (Eicosa-11,14-dienoic acid)	649	0.33 (±0.003)	0.67 (±0.05)
C20:3n-6 (Dihomo-γ-linolenic acid)	647	0.38 (±0.01)	0.88 (±0.03)
C20:4n-6 (Arachidonic acid)	649	2.42 (±0.04)	0.55 (±0.05)

N is the number of individuals with this fatty acid in the population. Relative content is the mean value of the content, and SE is the standard error. *h*^2^ is heritability.

## Data Availability

Not applicable.

## Supplementary Materials

The following supporting information can be downloaded at https://www.mdpi.com/article/10.3390/ani13203192/s1, Table S1: Detailed information on 25 fatty acids present in IMF of the *longissimus dorsi* of Ningxiang pigs; Table S2. Genome-wide significant SNPs loci for SFAs, MUFAs and PUFAs in Ningxiang pigs; File S1: GO and KEGG enrichment analysis; Figure S1. Manhattan plot for five SFAs (C14:0, C16:0, C17:0, C18:0, and C20:0). The x-axis represents the chromosomes, and y-axis represents the −log10 (*p*_value). The green line is genome-wide level threshold, the green dashed line is chromosome-level threshold; Figure S2. Manhattan plot for four MUFAs (C16:1, C18:1n-9(c), C18:1n-9(t), and C20:1). The x-axis represents the chromosomes, and y-axis represents the −log10 (*p*_value). The green line is genome-wide level threshold, the green dashed line is chromosome-level threshold; Figure S3. Manhattan plot for five PUFAs (C18:2n-6(c), C18:3n-3, C20:2, C20:3n-6, and C20:4n-6). The x-axis represents the chromosomes, and y-axis represents the −log10 (*p*_value). The green line is genome-wide level threshold, the green dashed line is chromosome-level threshold.
