# Supplementary material for: Genome-Wide Association Study and Identification of Candidate Genes for Intramuscular Fat Fatty Acid Composition in Ningxiang Pigs
Source: Animals (Basel). 2023 Oct 13;13(20):3192. doi: 10.3390/ani13203192 (PMC10603709; doi:10.3390/ani13203192)
Supplement: Supplementary file 1 [file animals-13-03192-s001.zip › Supp_Figure--Revised--1013.pdf]

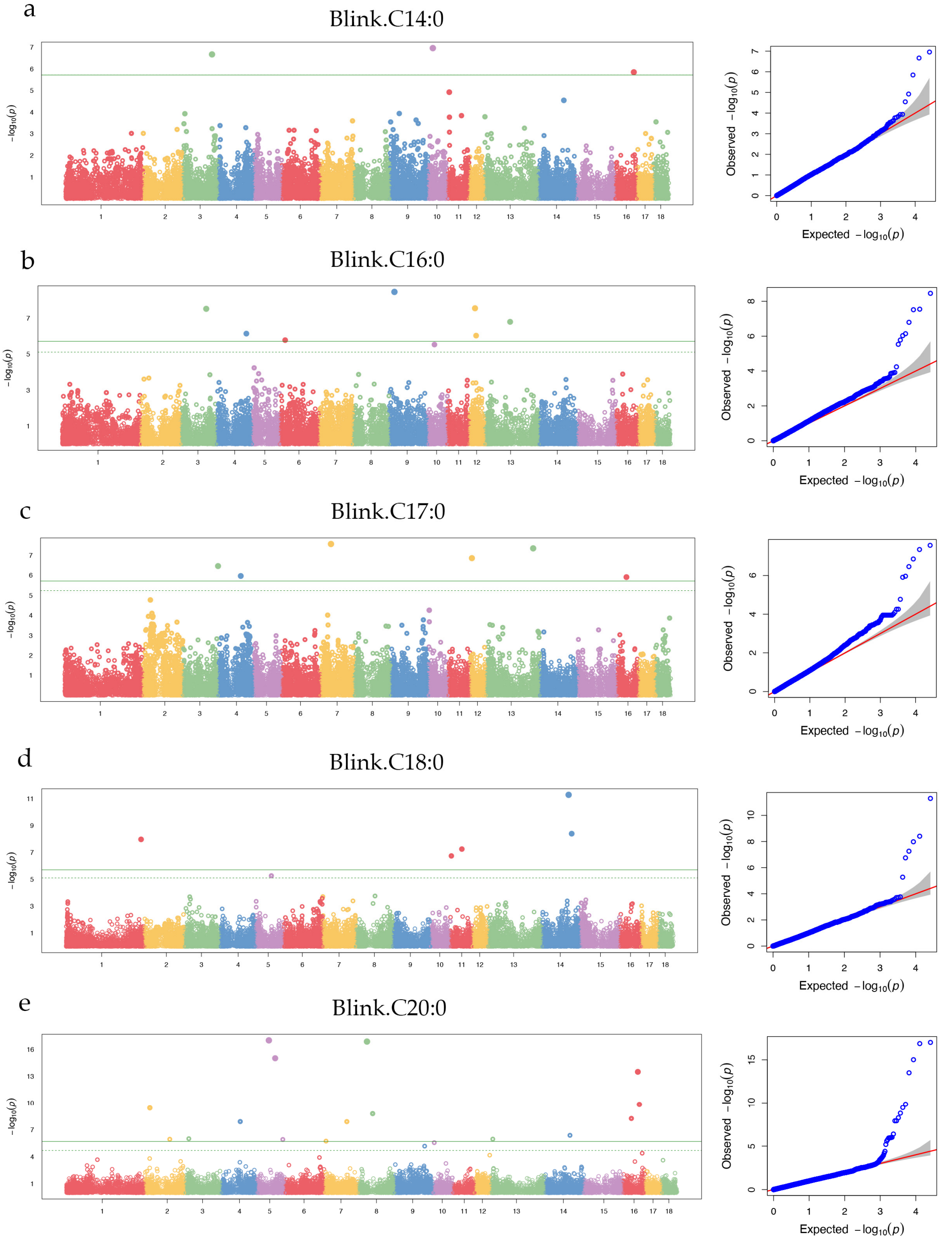

Figure S1. Manhattan plot for five SFAs (C14:0, C16:0, C17:0, C18:0, and C20:0). The x-axis represents the chromosomes, and y-axis represents the -log10 (*p_value*). The green line is genome-wide level threshold, the green dashed line is chromosome-level threshold.

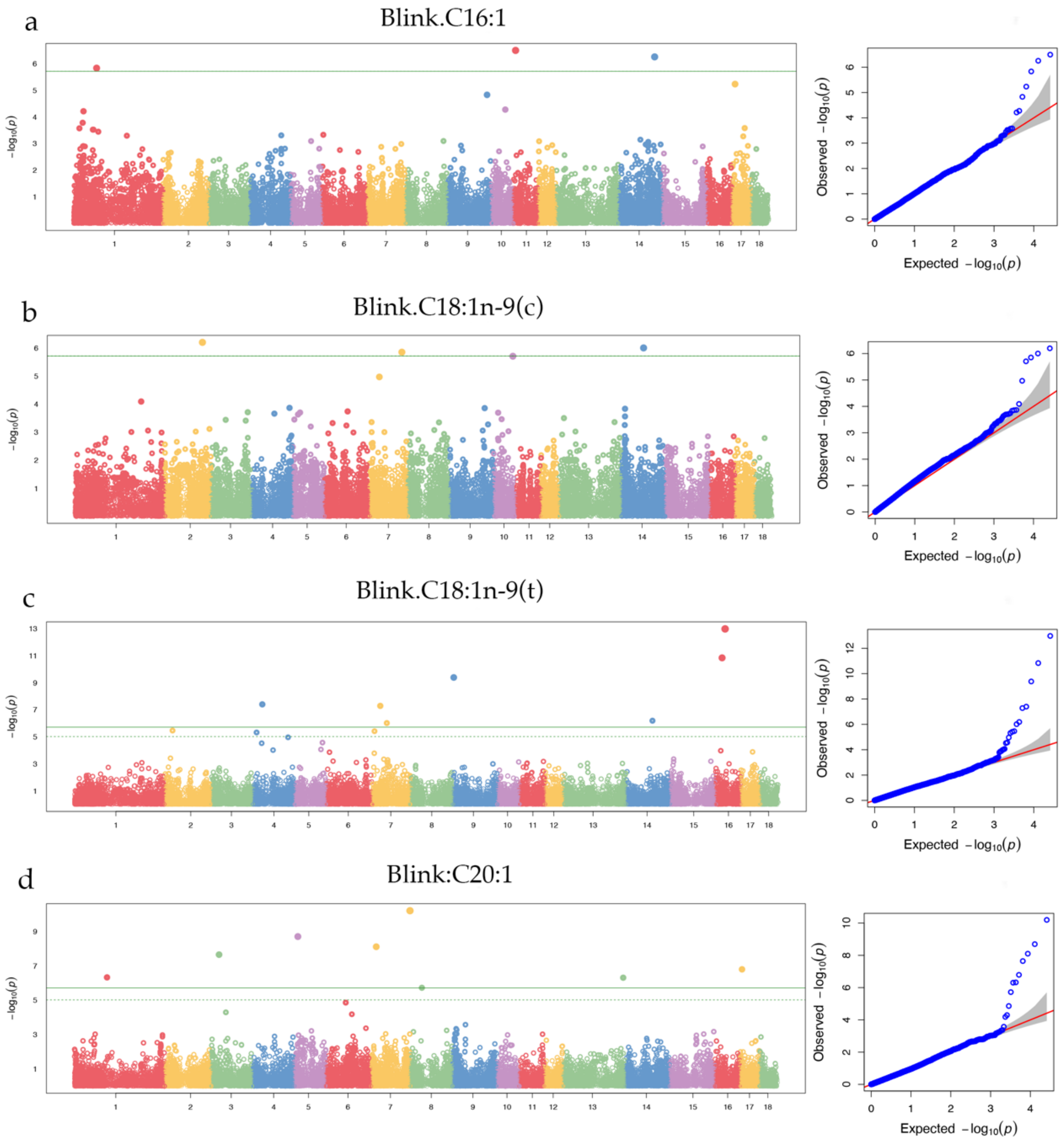

Figure S2. Manhattan plot for four MUFAs (C16:1, C18:1n-9(c), C18:1n-9(t), and C20:1). The x-axis represents the chromosomes, and y-axis represents the -log10 (p_value). The green line is genome-wide level threshold, the green dashed line is chromosome-level threshold.

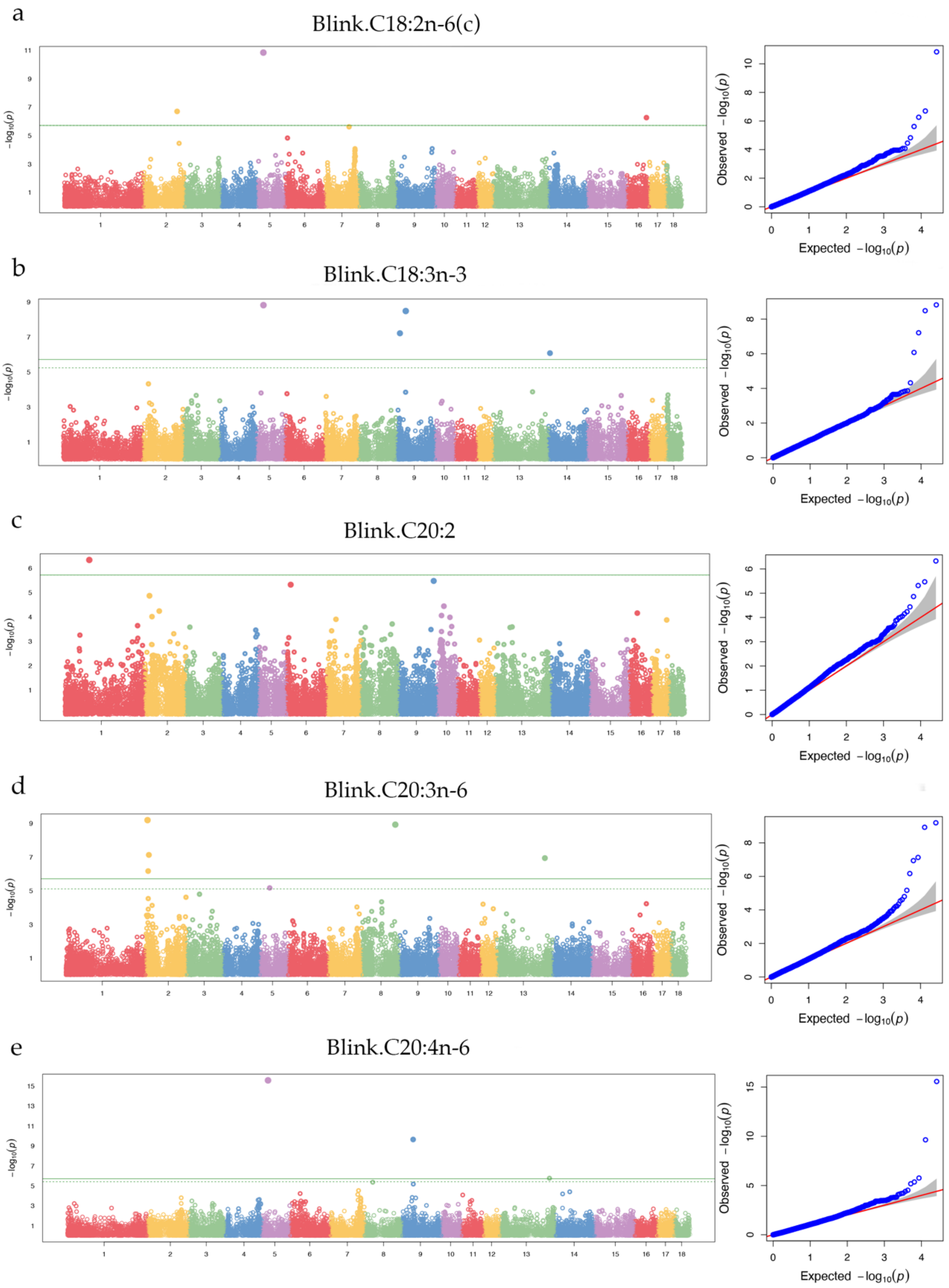

Figure S3. Manhattan plot for five PUFAs (C18:2n-6(c), C18:3n-3, C20:2, C20:3n-6, and C20:4n-6). The x-axis represents the chromosomes, and y-axis represents the -log10 (*p*_value). The green line is genome-wide level threshold, the green dashed line is chromosome-level threshold.
