# Supplementary material for: Genome-Wide Association Study and Identification of Candidate Genes for Intramuscular Fat Fatty Acid Composition in Ningxiang Pigs
Source: Animals (Basel). 2023 Oct 13;13(20):3192. doi: 10.3390/ani13203192 (PMC10603709; doi:10.3390/ani13203192)
Supplement: Supplementary file 1 [file animals-13-03192-s001.zip › Supp_Table--Revised--1013.pdf]

Table S1. Detailed information on 25 fatty acids present in IMF of the *longissimus dorsi* of Ningxiang pigs.

Substance Name	N	Relative Content (± SE) (%)
IMF (Intramuscular fat)	691	3.65 (± 0.04)
SFA (Saturated fat acid)	691	39.35 (± 0.38)
C10:0# (Capric)	279	0.24 (± 0.005)
C12:0# (Lauric acid)	428	0.12 (± 0.002)
C14:0 (Myristic acid)	649	1.42 (± 0.01)
C15:0# (Myristic oleic acid)	132	0.04 (± 0.002)
C16:0 (Palmitic acid)	651	26.38 (± 0.06)
C17:0 (Margaric acid)	645	0.15 (± 0.001)
C18:0 (Stearic acid)	651	13.42 (± 0.06)
C20:0 (Arachidic acid)	649	0.21 (± 0.002)
C22:0# (Behenic acid)	161	0.10 (± 0.004)
C23:0# (Tricosanoic acid)	201	0.17 (± 0.01)
C24:0# (Lignoceric acid)	191	0.20 (± 0.01)
MUFA (Monounsaturated fat acid)	691	41.88 (± 0.42)
C14:1# (Myristoleic acid)	80	0.04 (± 0.12)
C16:1 (Palmitoleic acid)	650	3.28 (± 1.03)
C17:1# (Margaroleic acid)	66	0.10 (± 0.004)
C18:1n-9(t) (Elaidic acid)	627	0.11 (± 0.001)
C18:1n-9(c) (Oleic acid)	651	40.28 (± 0.12)
C20:1 (Eicosenoic acid)	650	0.80 (± 0.01)
PUFA (Polyunsaturated fatty acid)	691	12.78 (± 0.18)
C18:2n-6(c) (Linoleic acid)	651	10.12 (± 0.10)
C18:3n-3 (α-Linolenic acid---ALA)	572	0.36 (± 0.01)
C18:3n-6#	220	0.07 (± 0.02)

(γ-Linolenic acid---GLA)		
C20:2 (Eicosa-11,14-dienoic acid)	649	0.33 (± 0.03)
C20:3n-3# (Dihomo-α-linolenic acid)	151	0.09 (± 0.02)
C20:3n-6 (Dihomo-γ-linolenic acid)	647	0.38 (± 0.01)
C20:4n-6 (Arachidonic acid)	649	2.42 (± 0.04)
C22:6n-3# (Docosahexaenoic acid---DHA)	64	0.09 (± 0.01)

N represents the number of individuals with this fatty acid detected in the population; relative content represents the mean value of the content, and SE represents the standard error; the "#" indicates the FA was removed due to a lower individual (N < 533).

Table S2. Genome-wide significant SNPs loci for SFAs, MUFAs and PUFAs in Ningxiang pigs.

Fatty acid	SNP	CHR	POS (bp)	MAF [1]	*p*-value	PVE (%) [2]	Nearest gene [3]	Location
C14:0	WU_10.2_3_116903421	3	116,903,421	0.10	2.15×10^{-7}	2.98	*ALK*	Intronic
	H3GA0053711	10	19,975,279	0.39	1.10×10^{-7}	4.21	*HNRNPU*	Intergenic
	ASGA0074106	16	75,024,639	0.20	1.43×10^{-6}	6.30	*MFAP3*	Intronic
C16:0	ALGA0020228	3	102,155,060	0.27	3.04×10^{-8}	0.96	*CAMKMT*	Intergenic
	WU_10.2_4_119395133	4	119,395,133	0.20	7.24×10^{-7}	0.71	*CEPT1*	Intronic
	ASGA0027821	6	20,665,813	0.08	1.66×10^{-6}	1.87	---	Intergenic
	MARC0001638	9	20,050,249	0.38	3.49×10^{-9}	1.54	---	Intergenic
	M1GA0024654	12	23,711,351	0.25	2.83×10^{-8}	1.49	*NPEPPS*	Intronic
	ASGA0053936	12	28,196,313	0.10	9.35×10^{-7}	2.46	*CA10*	Intergenic
	ALGA0071522	13	101,058,816	0.48	1.60×10^{-7}	0.92	*P2RY1* / *RAP2B*	Intergenic
C17:0	WU_10.2_3_142168876	3	142,168,876	0.22	3.47×10^{-7}	1.63	*ENSSSCG00000008655*	Intronic
	WU_10.2_4_89693248	4	89,693,248	0.13	1.09×10^{-6}	0.98	*ATP1B1* / *DPT*	Intergenic
	ALGA0040777	7	41,624,144	0.44	2.75×10^{-8}	1.06	*ENSSSCG00000027922*	Intergenic
	WU_10.2_12_7644839	12	7,644,839	0.02	1.40×10^{-7}	5.94	*SDK2*	Intergenic
	ASGA0059505	13	191,280,771	0.01	4.53×10^{-8}	11.39	*ENSSSCG00000012009*	Intergenic
	ASGA0097154	16	38,383,883	0.35	1.23×10^{-6}	0.74	*MIER3* / GPBP1	Intergenic
C18:0	ALGA0010606	1	302,716,687	0.02	1.05×10^{-8}	5.30	*SWI5*	3 prime UTR
	WU_10.2_11_3591593	11	3,591,593	0.45	1.78×10^{-7}	0.96	*CDK8*	Intronic
	DRGA0011206	11	45,910,375	0.02	5.51×10^{-8}	6.85	*KLHL1*	Intergenic
	WU_10.2_14_106229446	14	106,229,446	0.09	5.06×10^{-12}	6.06	*DKK1* / *PRKG1*	Intergenic
	ALGA0080940	14	118,552,421	0.30	3.94×10^{-9}	1.79	*AVPI1*	Intronic
C20:0	WU_10.2_2_19459316	2	19,459,316	0.01	3.53×10^{-10}	0.50	*U5* / *EXT2*	Intergenic
	MARC0046666	2	99,366,616	0.10	1.11×10^{-6}	0.04	*MEF2C*	Intergenic
	WU_10.2_3_13474115	3	13,474,115	0.17	9.65×10^{-7}	0.03	*SNORA79*	Intergenic
	ALGA0025658	4	75,387,791	0.03	1.21×10^{-8}	2.18	*ARMC1*	Intergenic
	DRGA0005776	5	46,748,561	0.01	9.76×10^{-18}	0	*IPO8*	Intergenic
	H3GA0016783	5	71,996,403	0.36	9.65×10^{-16}	0.12	*IL17RA* / *CECR2*	Upstream gene
	WU_10.2_5_102656712	5	102,656,712	0.01	1.17×10^{-6}	0.19	---	---
	WU_10.2_7_6641603	7	6,641,603	0.06	1.79×10^{-6}	0.04	*OFCC1*	Intergenic
	MARC0077077	7	90,165,851	0.03	1.22×10^{-8}	0.02	*U4*	---
	ALGA0047587	8	36,220,598	0.01	1.33×10^{-17}	89.85	---	---
	ALGA0049475	8	58,905,523	0.02	1.57×10^{-9}	0.38	*TMEM165* / *REST*	---
	WU_10.2_13_7954310	13	7,954,310	0.02	1.07×10^{-6}	0.13	*EFHB* / *PP2D1*	Intergenic
	H3GA0041501	14	98,921,568	0.37	4.07×10^{-7}	0.02	*ALOX5*	Intronic
	MARC0054269	16	33,826,642	0.45	5.43×10^{-9}	0.04	*ITGA1*	Intronic
	WU_10.2_16_59778879	16	59,778,879	0.12	3.17×10^{-14}	0.48	*SLIT3*	Intronic
	ASGA0073724	16	65,860,356	0.38	1.45×10^{-10}	0.06	---	---
C16:1	ALGA0004246	1	80,989,471	0.19	1.47×10^{-6}	8.55	*PREP*	Intergenic
	MARC0099145	11	7,302,533	0.26	3.19×10^{-7}	2.20	*ALOX5AP* / *MEDAG*	Intergenic
	ALGA0081341	14	127,218,925	0.42	5.58×10^{-7}	2.81	*U6*	Intergenic
C18:1n-9(c)	ALGA0015731	2	132,199,278	0.37	6.33×10^{-7}	0.67	*PRDM6*	Intronic
	WU_10.2_7_113985448	7	113,985,448	0.337	1.41×10^{-6}	1.67	*FLRT2*	Intergenic
	ASGA0064960	14	77,566,009	0.34	9.97×10^{-7}	0.41	*RUFY2*	Intronic
	H3GA0012422	4	31,763,547	0.02	4.00×10^{-8}	0.73	*RSPO2*	Intergenic
C18:1n-9(t)	DIAS0004691	7	29,798,220	0.02	5.20×10^{-8}	1.37	*RXRB* / *COL11A2*	Downstream gene
	ASGA0033619	7	52,577,418	0.02	9.68×10^{-7}	0.52	*MCM3* / *PAQR8*	---

	H3GA0025990	9	2,779,787	0.01	4.13×10^{-10}	2.69	*SYT9*	Intronic
	ALGA0079386	14	91,261,955	0.29	6.50×10^{-7}	0.26	*NRG3* / *SNORA31*	Intergenic
	H3GA0046208	16	22,351,887	0.01	1.46×10^{-11}	45.24	*CAPSL*	Intronic
	DRGA0016063	16	32,872,449	0.04	1.06×10^{-13}	31.52	*ISL1*	Intergenic
	ASGA0004095	1	113,473,890	0.24	4.76×10^{-7}	0.79	*ENSSSCG00000004527*	Intergenic
	ASGA0085560	3	26,316,304	0.36	2.27×10^{-8}	1.43	*GP2 /GPR139*	Intergenic
	WU_10.2_5_13180559	5	13,180,559	0.38	2.03×10^{-9}	0.85	*CRY1*	Intronic
C20:1	ASGA0031521	7	17,152,543	0.02	7.91×10^{-9}	6.73	*CDKAL1*	---
	7_134762912	7	134,762,912	0.26	6.37×10^{-11}	1.67	*SCARNA6*	Intergenic
	ALGA0111031	8	41,395,488	0.25	1.91×10^{-6}	2.27	*USP46*	Intergenic
	ASGA0059943	13	210,282,758	0.10	4.97×10^{-7}	0.82	*CLDN14* / *SIM2*	Intergenic
	H3GA0047778	17	7,610,059	0.03	1.62×10^{-7}	3.49	---	---
	ALGA0015731	2	132,199,278	0.37	1.99×10^{-7}	2.10	*CEP120*	Intronic
C18:2n-6(c)	WU_10.2_5_24153921	5	24,153,921	0.02	1.45×10^{-11}	18.22	*RDH16* / *NDUFA4L2*	Intergenic
	ASGA0085192	16	76,899,854	0.05	5.47×10^{-7}	6.33	*ENSSSCG00000017077*	Intergenic
	MARC0014875	5	26,784,432	0.01	1.52×10^{-9}	21.92	*SLC16A7*	Intergenic
	ALGA0111381	9	14,991,770	0.02	6.15×10^{-8}	5.51	*ssc-mir-708*	Intergenic
C18:3n-3	DRGA0009320	9	37,972,729	0.08	3.28×10^{-9}	16.44	*ENSSSCG00000014993*	Intronic
	ASGA0060681	14	5,504,363	0.42	8.41×10^{-7}	1.53	*SNORA25*	Intergenic
C20:2	ALGA0004743	1	96,303,394	0.08	4.70×10^{-7}	10.78	*ENSSSCG00000022379*	Intergenic
	WU_10.2_2_7173969	2	7,173,969	0.23	6.35×10^{-10}	2.14	*FLRT1*	Intronic
	WU_10.2_2_9630034	2	9,630,034	0.19	6.76×10^{-7}	2.15	*SDHAF2*	Missense
C20:3n-6	ALGA0106081	2	12,459,648	0.18	7.40×10^{-8}	1.62	*ENSSSCG00000022943*	Upstream gene
	ASGA0039809	8	130,628,617	0.18	1.18×10^{-9}	1.84	*EIF4E*	Intronic
	ALGA0115480	13	187,857,350	0.11	1.14×10^{-7}	1.57	*ROBO2*	Intergenic
	ALGA0031262	5	23,686,043	0.01	2.71×10^{-16}	31.76	*PTGES3*	Intronic
C20:4n-6	WU_10.2_9_42529952	9	42,529,952	0.02	2.28×10^{-10}	7.56	*C11orf87* / *U1*	Intergenic
	WU_10.2_13_194176619	13	194,176,619	0.22	1.73×10^{-6}	1.20	*U6*	Intergenic

[1] MAF: minor allelic frequency;

[2] PVE: explained phenotype variance;

[3] The nearest genes are the closest genes within 500kb upstream and downstream of the significant SNP.
